# Highlights from Heart Rhythm 2018: Atrial Fibrillation

**DOI:** 10.19102/icrm.2018.090907

**Published:** 2018-09-15

**Authors:** Rahul N. Doshi

**Affiliations:** ^1^Division of Cardiology, Keck School of Medicine, University of Southern California, Los Angeles, CA, USA

**Keywords:** Atrial fibrillation, commentary, heart rhythm society, review

Earlier this year in Boston, the world of cardiac rhythm management was focused on the annual Scientific Sessions of the Heart Rhythm Society. I think it is fair to say that atrial fibrillation (AF) ablation stole the show. With the presentation of the Catheter Ablation versus Antiarrhythmic Drug Therapy for AF (CABANA) trial, the most anxiously anticipated trial in recent years, and many other important contributions from large multicenter trials, catheter ablation for AF was at the center of the cardiology world. Let us get right to it.

## The CABANA trial

Douglas Packer, MD, of the Mayo Clinic, presented the primary results from the CABANA trial to an overflowing crowd during the first late-breaking clinical trials session.^1^ As most of you are aware, this trial was a 1:1 randomization of state-of-the-art ablation versus drug therapy as first-line treatment for any AF requiring treatment. Prior studies demonstrating the efficacy of catheter ablation have involved patients who failed prior drug therapy; hence, the current consensus document recommendation.^2^ The primary endpoint of CABANA was a composite score of all-cause mortality, disabling stroke, serious bleeding, or cardiac arrest. The predefined secondary endpoints were all-cause mortality and a combination of death and cardiovascular hospitalization. In the study, 2,204 patients were randomized, which, of course, is an incredible achievement. However, there was a very high crossover rate, with 27% of the patients who were randomized to drug therapy ultimately receiving an ablation. There were 42% paroxysmal, 47% persistent, and 10% longstanding cases of AF included. One-third of these patients had a history of heart failure, whereas 10% had a history of stroke or transient ischemic attack (TIA). The average follow-up was 48 months, with approximately 90% of patients completing follow-up.

Drum roll, please….

In the study, there were no significant differences between the two arms in the intention-to-treat analysis for the primary composite endpoint (8.0% ablation versus 9.2% drug therapy; p = 0.30), nor were there differences among any of the individual components of the composite endpoint. Additionally, there was no difference between the two groups with respect to all-cause mortality (5.2% ablation versus 6.1% drug therapy; p = 0.38). There was, however, a statistically significant difference in the prespecified composite endpoint of death and cardiovascular hospitalization (51.7% versus 58.1%; p = 0.001). This constitutes a 17% reduction in this composite endpoint. Consistent with prior studies, AF-free survival was superior in the ablation group at four years in approximately 60% in the ablation arm versus 40% in the drug treatment group.

In the analysis of actual treatment received (recall the high crossover rate), there were significant differences in both the primary endpoint and the secondary endpoints, with ablation appearing to be superior to antiarrhythmic drug treatment. For the composite primary endpoint, 7.0% of patients receiving ablation reached the endpoint versus 10.9% in the drug arm (p = 0.006). The all-cause mortality was 4.4% for patients undergoing ablation versus 7.5% for patients receiving drug treatment (p = 0.005). Stated differently, there was a 41% relative risk reduction in mortality in patients undergoing catheter ablation in comparison with the group receiving drug treatment for AF. For the composite endpoint of death and cardiovascular hospitalization, 41.2% of patients experienced an event in the ablation group versus 74.9% in the drug arm (p = 0.002). Furthermore, there appears to be a very low price to pay for ablation with regards to safety: only 0.8% of patients had cardiac tamponade requiring intervention and 0.3% of patients experienced periprocedural TIA. No atrioesophageal fistulas were seen.

So, where does this leave us?

Dr. Packer’s conclusion, of course, was that there was no benefit seen in the composite endpoint or in total mortality but rather only in the composite endpoint of death and cardiovascular hospitalization. This was explained by the very high crossover rate and the lower than anticipated event rate. He did state that ablation was clearly more effective at reducing AF and that there was a significant mortality benefit seen in patients who underwent ablation (complete with all the biases that arise from this analysis).

Thus, it is fair to say that patients who undergo ablation for whatever reason as first-line treatment do better than those who are treated with antiarrhythmic drug therapy. The “purist” or “trialist” will correctly point out that we cannot say ablation is superior to drug therapy as an initial treatment of atrial fibrillation and there of course has also been much speculation as to whether a study with a sham procedure arm is necessary to demonstrate that catheter ablation of AF is superior to drug therapy. However, it might be prudent to think back to similar debates in our own field.

The debate generated here seems very reminiscent of the days of implantable cardioverter-defibrillator (ICD) trials for primary prevention before the Suden Cardiac Death in Heart Failure (SCDHeFT) trial was presented. This study was definitive, but trial after trial suggested the superiority of the ICD as compared with antiarrhythmic treatment for patients with low ejection fractions and heart failure—including trials that were not designed to be ICD trials [remember the Multicenter Unsustained Tachycardia Trial (MUSTT)?]. We all should remember the difficulties in enrolling patients for primary prevention trials before SCDHeFT, given the growing body of literature supporting the use of the ICD for primary prevention in high-risk patients. Many participants in CABANA expressed similar extreme difficulties in enrolling patients for the trial, and several just stopped enrolling. Dr. Packer and the CABANA investigators should be congratulated for bringing this sentinel trial, for all its limitations, to completion. In addition, I think that, as electrophysiologists, we can look at other concepts that demonstrate the importance of catheter ablation in the treatment of AF.

## Atrial fibrillation burden in CASTLE-AF

A perfect example of changing concepts on how we analyze patients undergoing catheter ablation was presented during the same session as that which contained the CABANA trial. Johannes Brachman, MD, of Coburg Hospital in Germany, on behalf of the Catheter Ablation versus Standard Conventional Treatment in Patients With Left Ventricular Dysfunction and AF (CASTLE-AF) study investigators, presented data analysis from the CASTLE-AF trial and the benefits of reducing AF burden.^3^ The CASTLE-AF trial, published earlier this year,^4^ demonstrated a significant benefit in the primary composite endpoint of death or heart failure hospitalization in patients with AF and heart failure who underwent ablation as compared with using conventional therapy. All of these patients had an implanted ICD and, thus, AF recurrence and burden could be accurately assessed via remote monitoring. As has been previously demonstrated, catheter ablation was effective in this population at reducing both time to first recurrence of AF and AF burden. In the trial, a high AF burden predicted the primary endpoint as well as total mortality. However, the investigators demonstrated that a reduction in AF burden but not time to first recurrence impacted both the composite endpoint and mortality in this high-risk population. This is a very different way of looking at treating AF successfully, analogous to pacing burden and the precipitation of heart failure or mortality in the cardiomyopathy population (I, for one, will not be sad about not having to generate Kaplan–Meier curves). It certainly raises a consideration of the benefits of catheter ablation as a “treatment” versus thinking of ablation solely as “curative” therapy, or considering a procedure to be a failure based solely on a recurrence.

## Ablation technologies

### Pulsed electrical field ablation for pulmonary vein isolation

Elsewhere at the show, Reddy et al.^5^ presented a “first-in-man” experience with pulsed electrical field ablation for AF. This technology involves the delivery of millisecond pulses of very-high-voltage bipolar energy to create nonthermal ablation, potentially eliminating the risk of both thrombus formation and collateral injury to the surrounding structures. In this first experience, the authors reported data from 15 patients employing an endocardial catheter and from seven patients treated with an epicardial system during sternotomy developed by Iowa Approach (Iowa City, IA, USA). They report acute procedural success in all 15 patients undergoing endocardial ablation and six of the seven patients undergoing the epicardial procedure. There was one failure in the epicardial group secondary to equipment failure. They reported acutely very short ablation times and no safety issues in this small initial experience. This represents a very exciting technology that of course needs to be validated further, especially regarding long-term durability; still, these results suggest a potential decrease in risks associated with radiofrequency ablation and a shortening of procedure times.

## Alternative treatments

### Long-term reduction in atrial fibrillation burden with epicardial botulinum toxin

Romanov et al.^6^ reported on the three-year data from their randomized cohort of patients undergoing coronary artery bypass grafting surgery injected with epicardial botulinum toxin. They have previously published 12-month follow-up data showing a significant reduction of AF burden in a low-burden paroxysmal AF population (0.3% treated with botulinum versus 1.7% for placebo; p = 0.003).^7^ Their primary endpoint was the incidence of atrial arrhythmia lasting more than 30 seconds in duration as recorded by an implantable cardiac monitor while off antiarrhythmic drug therapy. Each arm of the study had 30 patients. The authors reported a 23% incidence of any arrhythmia at three years versus 50% in the placebo group (p = 0.02). This corresponds to a significant reduction in AF burden (1.3% versus 6.9%; p = 0.007) and resulted in decreased hospitalizations and antiarrhythmic drug use. This represents the first demonstration of a long-term benefit of autonomic modulation therapy for the treatment of AF. Although the authors’ state that the precise mechanism of this effect is unknown, as they have previously demonstrated that the measurable autonomic effect lasts about three months, they call attention to the difference in this therapy as an “immunomodulating” versus “immunodestructive” option such as catheter ablation or ganglia plexi denervation. Further investigation is clearly needed for this very exciting therapy. This is also another example of examining AF burden as compared to first recurrence.

## Thromboembolism

### Risk of device-related thrombus with left atrial occlusion

Reddy et al.^8^ presented a meta-analysis of the incidence of device-related thrombus (DRT) seen with use of the WATCHMAN™ left atrial appendage (LAA) occlusion (LAAO) device (Boston Scientific, Natick, MA, USA). They looked at data from four United States Food and Drug Administration–approved clinical trials that included more than 1,700 successful implants. They found a total incidence of 3.7% of DRT in the pooled analysis. This finding is similar to that seen with previous data for patients receiving this device.^9^ Several factors where associated with the risk of DRT, such as CHA_2_DS_2_-VASc score, prior stroke/TIA, left ventricular ejection fraction, permanent AF, and the size of the LAA. The presence of DRT was associated with an approximately fourfold increase in the risk of stroke. Moreover, the study calls into question whether or not we should routinely perform transesophageal echocardiography surveillance at six months after surgery or whether we should aggressively monitor only the high-risk patients with significant risk factors. Thus, optimal surveillance strategies have yet to be determined but are an important consideration given the significant incidence.

### Compliance with direct oral anticoagulant therapy

A significant criticism of LAAO use is the lack of direct randomized comparison with direct oral anticoagulants (DOACs). Much of this is centered on the potential for better outcomes with DOACs as compared with using vitamin K antagonists (VKAs). In their presentation, Lakkireddy et al.^10^ raised questions regarding improved outcomes with DOAC therapy as compared with VKA therapy. They analyzed health claims data using the IBM Watson Health MarketScan^®^ application (IBM Corp., Armonk, NY, USA). In a study cohort of more than 80,000 patients receiving oral anticoagulant therapy for nonvalvular AF and a CHA_2_DS_2_-VASc score ≥ 2, they found a slight increase in thromboembolic events in patients treated with a DOAC versus a VKA (3.37 events versus 3.16 events per 100 patient-years) and an equivalent stroke rate (3.73 events per 100 patient-years). The bleeding risk was lower in the DOAC group including with regard to hemorrhagic stroke (0.54 events versus 0.88 events per 100 patient-years). Although a higher number of patients remained adherent to therapy in the DOAC group, adherence was still poor, with up to one-third of patients not remaining on therapy. Importantly, patients with poor adherence to DOAC therapy had the highest stroke risk of any group. This study demonstrates the limitations of medical therapy in a “real-world” database and highlights the importance of implementing mechanisms that improve adherence or that do not rely on patient compliance (such as LAAO).

## Putting it all together

Kita et al.^11^ presented the one-year data of patients with persistent/longstanding persistent AF who underwent AF ablation with LAA electrical isolation together with LAAO. The group previously reported acute procedural success, demonstrating the feasibility of a concomitant approach.^11^ All patients were recommended to remain on anticoagulation therapy for three months postprocedure and then switch to antiplatelet therapy. In a small cohort of patients including both first-time and redo ablation cases, the investigators found an AF-free survival rate for any recurrence lasting more than 30 seconds of 80% at one year, with all patients mostly in sinus rhythm. There was no evidence of thromboembolism. One DRT was seen in a patient with premature cessation of DOAC therapy. These data support the importance of LAA electrical isolation in persistent AF as previously demonstrated^12^ but also potentially eliminate the risk of thrombus formation post-LAA isolation. Larger randomized trials are needed to fully test this hypothesis.

## Conclusions

The 2018 Annual Scientific Sessions of the Heart Rhythm Society will be remembered as a pivotal time in that the efficacy of catheter-based interventions for the treatment of AF was demonstrated. However, the research presented still leaves pending questions and illustrates the need for further ground-breaking science. We will all anxiously await next year!
